# A novel set of vectors for Fur-controlled protein expression under iron deprivation in *Escherichia coli*

**DOI:** 10.1186/s12896-016-0298-1

**Published:** 2016-09-13

**Authors:** Paknoosh Pakarian, Peter D. Pawelek

**Affiliations:** 1 Department of Chemistry and Biochemistry, Concordia University, 7141 Sherbrooke St., W., Montreal, QC H4B 1R6 Canada; 2 Groupe de Recherche Axé sur la Structure des Protéines (GRASP), Montreal, Canada

**Keywords:** Ferric uptake regulator, Protein expression, Bidirectional promoter, Bacterial two-hybrid, CAS assay

## Abstract

**Background:**

In the presence of sufficient iron, the *Escherichia coli* protein Fur (Ferric Uptake Regulator) represses genes controlled by the Fur box, a consensus sequence near or within promoters of target genes. De-repression of Fur-controlled genes occurs upon iron deprivation. In the *E. coli* chromosome, there is a bidirectional intercistronic promoter region with two non-overlapping Fur boxes. This region controls Fur-regulated expression of *entCEBAH* in the clockwise direction and *fepB* in the anticlockwise direction.

**Results:**

We cloned the *E. coli* bidirectional *fepB*/*entC* promoter region into low-copy-number plasmid backbones (pACYC184 and pBR322) along with downstream sequences encoding epitope tags and a multiple cloning site (MCS) compatible with the bacterial adenylate cyclase two-hybrid (BACTH) system. The vector pFCF1 allows for iron-controlled expression of FLAG-tagged proteins, whereas the pFBH1 vector allows for iron-controlled expression of HA-tagged proteins. We showed that *E. coli* knockout strains transformed with pFCF1-*entA*, pFCF1-*entE* and pFBH1-*entB* express corresponding proteins with appropriate epitope tags when grown under iron restriction. Furthermore, transformants exhibited positive chrome azurol S (CAS) assay signals under iron deprivation, indicating that the transformants were functional for siderophore biosynthesis. Western blotting and growth studies in rich and iron-depleted media demonstrated that protein expression from these plasmids was under iron control. Finally, we produced the vector pFCF2, a pFCF1 derivative in which a kanamycin resistance (*KanR*) gene was engineered in the direction opposite of the MCS. The *entA* ORF was then subcloned into the pFCF2 MCS. Bidirectional protein expression in an iron-deprived pFCF2-*entA* transformant was confirmed using antibiotic selection, CAS assays and growth studies.

**Conclusions:**

The vectors pFCF1, pFCF2, and pFBH1 have been shown to use the *fepB*/*entC* promoter region to control bidirectional *in trans* expression of epitope-tagged proteins in iron-depleted transformants. In the presence of intracellular iron, protein expression from these constructs was abrogated due to Fur repression. The compatibility of the pFCF1 and pFBH1 backbones allows for iron-controlled expression of multiple epitope-tagged proteins from a single co-transformant.

**Electronic supplementary material:**

The online version of this article (doi:10.1186/s12896-016-0298-1) contains supplementary material, which is available to authorized users.

## Background

Bacterial iron acquisition is tightly controlled in order to ensure adequate iron uptake to support cellular survival and growth while preventing an over-accumulation of iron leading to oxidative damage [1]. To promote iron homeostasis, most genes involved in iron uptake mechanisms are only abundantly expressed under conditions of low intracellular iron, and are typically repressed when the cell is replete with iron. In *Escherichia coli*, one of the major regulators of iron homeostasis is the protein Fur (Ferric Uptake Regulator), a homodimeric protein with 17 kDa subunits [2–4]. Given its central role in regulating iron homeostasis and oxidative stress, Fur, along with the small RNA RyhB, are known virulence factors in a number of pathogenic bacterial species that require iron from host organisms [5]. Fur, in the iron-bound *holo* form, binds tightly to a recognition site known as the Fur box. Although there is variation in Fur box sequences, they all share identity with a 19-bp consensus sequence 5′-(GATAATGAT(A/T)ATCATTATC)-3′ [6, 7]. In addition to its classical role as a repressor, *holo*-Fur has been reported to activate a number of gene targets [8, 9]. A recent genome-wide study has also reported that Fur regulates 82 genes in *E. coli*, both by *apo*- and *holo*-Fur activation and *holo*-Fur repression [10]. In the classical *holo*-Fur repression mechanism, iron-bound Fur binds to a Fur box sequence that overlaps with, or is proximal to, promoters of iron-responsive genes, thus preventing their transcription [11]. When intracellular iron is depleted, Fe^2+^ is released from Fur, causing conformational changes in the protein resulting in dissociation from the Fur box [12]. This de-repression results in the up-regulation of Fur-controlled genes. Numerous genes are controlled by *holo*-Fur, including those that encode: (*i*) proteins involved in siderophore-mediated iron uptake [13], (*ii*) small RNAs such as *ryhB* that regulate bacterial iron uptake [14], (*iii*) some TCA cycle enzymes [15], (*iv*) superoxide dismutase [14, 16], and (*v*) Fur itself [17].

**Fig. 1:**
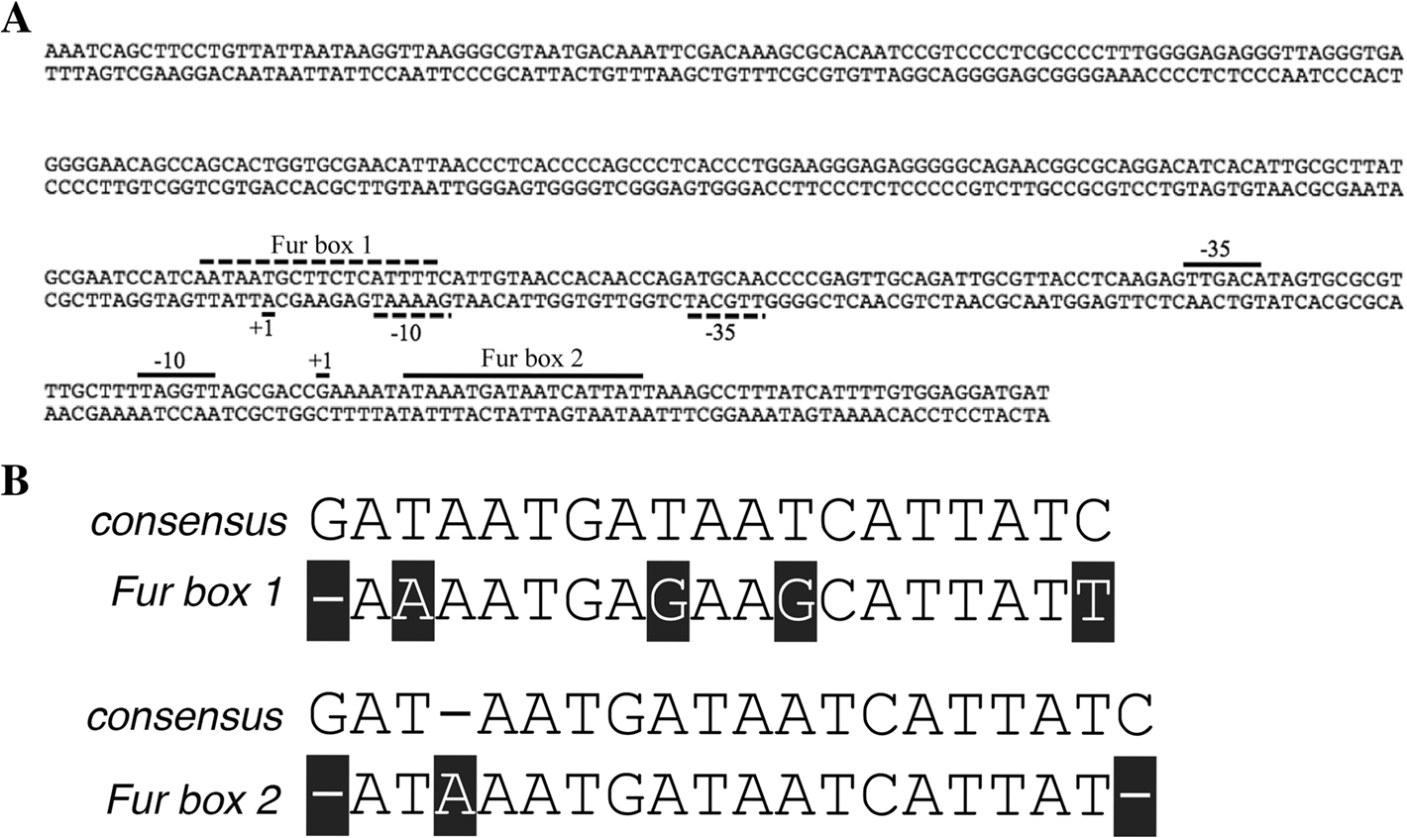
The intercistronic bidirectional promoter region between *E. coli fepB* and *entC*. **a** The sequence contains all nucleotides between the *fepB* and *entC* start codons (624511–624884 in *E. coli* K12 MG1655 (NCBI Reference Number: NC_000913.3). Positions of anti-clockwise regulatory elements (Fur box1, +1 − 10 and −35 sequences for *fepB* transcriptional regulation) are indicated by dashed lines. Positions of clockwise regulatory elements (Fur box 2, +1 − 10 and −35 sequence for *entCEBAH* transcriptional regulation) are shown as solid lines. Fur box 1 and Fur box 2 were identified previously [18, 19]. **b** Sequence alignment of the Fur box consensus sequence with Fur box 1 and Fur box 2. Positions diverging from the consensus sequence are highlighted

Here we report a novel set of vectors that contain the *E. coli fepB*-*entC* promoter region that has two bidirectional Fur box sequences (Fig. 1a) identified from previous studies [18–20]. Fur box 1 (5′-AAAATGAGAAGCATTATT-3′) and Fur box 2 (5′-ATAAATGATAATCATTAT-3′) differ from the consensus sequence by 5 and 3 nucleotides, respectively (Fig. 1b). When incorporated into the vectors, this region controls plasmid-borne protein expression by Fur de-repression upon iron restriction. They can be used for iron-controlled expression of any subcloned ORF, even those not typically found under Fur control. We designed these vectors for expression of epitope-tagged proteins using an MCS compatible with the bacterial adenylate cyclase two-hybrid (BACTH) system, allowing for subcloning of ORFs of interest from a BACTH system to the Fur-controlled protein expression system reported here. Proteins expressed from these vectors contain cleavable N-terminally fused epitope tags (FLAG or HA) that are useful for various immunochemical approaches.

## Methods

### Reagents, plasmids, software and primers

All reagents were purchased from Bioshop Canada, Inc. (Burlington, Ontario) unless otherwise indicated. Plasmids used in this study are summarized in Table 1. All plasmid maps were generated using SnapGene® Viewer (GSL Biotech; http://www.snapgene.com). All primer sequences used in this study are found in Additional file 1: Table S1.

**Table 1:** Plasmids used in this study

Plasmid name	Resistance	Source
pACYC184	Cm^R^, Tet^R^	NEB
pBR322	Amp^R^, Tet^R^	NEB
pUT18C	Amp^R^	Euromedex
pKT25	Kan^R^	Euromedex
pFCF1	Tet^R^	This study
pFBH1	Amp^R^	This study
pFCF2	Tet^R^, Kan^R^	This study

*NEB* New England Biolabs

*Amp* ^*R*^ ampicillin resistance, *Cm* ^*R*^ chloramphenicol resistance, *Kan* ^*R*^ kanamycin resistance, *Tet* ^*R*^ tetracycline resistance

### Production of pFCF1 and pFCF2

A 489-bp DNA fragment (gBlock1; Additional file 2: Figure S1) with flanking NcoI and EcoRI sites containing: (*i*) the *E. coli fepB*/*entC* bidirectional promoter region, (*ii*) the FLAG tag sequence, (*iii*) the TEV protease cleavage site sequence, and (*iv*) the multiple cloning site (MCS) from pUT18C (Euromedex) was synthesized as a gBlock® (Integrated DNA Technologies, San Diego, California). This fragment was digested with NcoI and EcoRI (NEB) and cloned into pACYC184 linearized with the same restriction enzymes. The resulting vector was named pFCF1. In order to create pFCF1-*entA*, pFCF1-*entE*, and pFCF1-*T25*, *E. coli entA* and *entE* ORFs were PCR-amplified from pCA24N-based constructs as reported previously [21]. The *B. pertussis* T25 fragment was PCR-amplified from pKT25 (Euromedex). PCR products were subcloned into the KpnI and EcoRI sites of the pFCF1 MCS.

A 903-bp DNA fragment (gBlock2; Additional file 3: Figure S2) containing: (*i*) the HA tag sequence and (*ii*) the kanamycin resistance (*KanR*) gene from pKT25 (Euromedex) was synthesized as a gBlock® (Integrated DNA Technologies, San Diego California). The ends of this fragment contained ~40-nucleotide regions that overlapped with corresponding sequences upstream and downstream of NcoI and ScaI sites, respectively, in pFCF1. The synthesized fragment was inserted into pFCF1 digested with NcoI and ScaI using the Gibson Assembly Master Mix (New England Biolabs) according to manufacturer’s protocol. The resulting vector was named pFCF2. The *E. coli entA* ORF was subcloned between KpnI and EcoRI sites of digested pFCF2 to generate pFCF2-*entA*. pFCF1 and pFCF2 constructs were verified by DNA sequencing (McGill University and Génome Québec Innovation Centre).

### Production of pFBH1

A 522-bp DNA fragment (gBlock3; Additional file 4: Figure S3) containing: (*i*) the *E. coli fepB*/*entC* bidirectional promoter region, (*ii*) the HA tag sequence, (*iii*) the TEV protease cleavage site sequence, and (*iv*) the multiple cloning site (MCS) from pUT18C (Euromedex) was synthesized as a gBlock® (Integrated DNA Technologies, San Diego California). The ends of this fragment contained 25-nucleotide regions that overlapped with corresponding sequences upstream and downstream of EcoRI and SalI sites, respectively, in pBR322. The fragment was inserted into pBR322 digested with EcoRI and SalI using the Gibson Assembly Master Mix (New England Biolabs) according to the manufacturer’s protocol. The resulting vector was named pFBH1. The *E. coli entB* ORF was subcloned into the KpnI and EcoRI sites of the pFBH1 MCS. The pFBH1 construct was verified by DNA sequencing (McGill University and Génome Québec Innovation Centre).

### CAS assays

All plasmid constructs and empty vector controls were transformed into respective *E. coli* BW25113 (F^−^, *Δ(araD-araB)567, ΔlacZ4787(::rrnB-3), λ*^*−*^*, rph-1, Δ(rhaD-rhaB)568, hsdR514*) knockout strains [22] that have been modified to remove the kanamycin resistance gene as reported previously [21]. Strains transformed with, pFCF1, pFCF1-*entA*, and pFCF1-*entE* were plated onto LB agar containing 12.5 μg/ml tetracycline. Strains transformed with pFCF2, pFCF2-*entA* were plated onto LB agar containing 12.5 μg/ml tetracycline and 50 μg/ml kanamycin. Strains transformed with pFBH1 or pFBH1-*entB* were plated onto LB agar containing 100 μg/ml ampicillin. All plates were incubated overnight at 37 °C. Overnight cultures (LB broth with appropriate antibiotic) from colony picks were diluted 1:1000 in 1× modified M9 medium [21] and 12.5 μg/ml tetracycline with or without 50 μg/ml kanamycin or 100 μg/ml ampicillin. Minimal medium cultures were grown at 37 °C overnight. CAS-agar plates supplemented with appropriate antibiotics were prepared according to Payne et al. [23]. CAS plates were spotted with 1 μL overnight cultures and incubated at 37 °C for approximately 16 h. Presences of orange halos were indicative of enterobactin biosynthesis [24]. Each CAS assay was performed in triplicate.

### Growth studies

Single colony picks of transformants used for CAS assays were used to inoculate LB broth supplemented with appropriate antibiotics. Overnight cultures were diluted 1:100 in LB plus antibiotics and then grown at 30 °C until they reached an A_600_ of 1.00. Cultures were centrifuged for 1 min at 21,000 × g and cell pellets were resuspended in 1× modified M9 medium such that all cultures were diluted to an equivalent cell density (A_600_ = 1.00). Cultures for growth measurements were then prepared by 1:1000 dilution into 1× modified M9 medium plus 50 μM 2,2′-dipyridyl containing appropriate antibiotic. Diluted cultures were incubated at 30 °C for 16 h with agitation. Cell densities were measured as A_600_ values. Growth experiments were performed in triplicate.

Additional growth studies of pFCF2-*entA* transformants were performed to demonstrate that the kanamycin resistance gene in this construct was under Fur control. Colony picks from the *E. coli* BW25113 *entA*^*−*^ strain transformed with pFCF2-*entA* were used to inoculate 3 ml of LB broth containing 12.5 μg/ml tetracycline and 50 μg/ml kanamycin. Overnight cultures incubated at 30 °C were diluted 1:100 in LB broth containing 12.5 μg/ml tetracycline and 50 μg/ml kanamycin and then grown at 30 °C until an A_600_ of 1.0 was reached. Cultures were centrifuged for 1 min at 21,000 × g and cell pellets were resuspended in 1× modified M9 medium such that all were diluted to an equivalent cell density (A_600_ = 1.00). Cultures for growth measurements were then prepared by 1:1000 dilution into one of the following: (*i*) 1× modified M9 medium plus 50 μM 2,2′-dipyridyl containing 12.5 μg/ml tetracycline, (*ii*) 1× modified M9 medium plus 50 μM 2,2′-dipyridyl containing 12.5 μg/ml tetracycline and 50 μg/ml kanamycin, (*iii*) LB broth containing 40 μM FeSO_4_, 12.5 μg/ml tetracycline, 0.2 % glucose, and (*iv*) LB broth containing 40 μM FeSO_4_, 12.5 μg/ml tetracycline and 50 μg/ml kanamycin, 0.2 % glucose. Diluted cultures were incubated at 30 °C for 16 h with agitation. Cell densities were measured as A_600_ values. Growth experiments were performed in triplicate.

### Western blotting

Expression constructs were transformed into competent *E. coli* BW25113 cells. Single colony picks of transformants were used to inoculate LB broth supplemented with appropriate antibiotics. Overnight cultures were diluted 1:100 in LB plus antibiotics and then grown at 30 °C until they reached an A_600_ of 1.0. Cultures were centrifuged for 1 min at 21,000 × g and cell pellets were resuspended in 1× modified M9 medium and then diluted to an equivalent cell density (A_600_ = 1.00). Cultures were prepared by 1:1000 dilution into iron-depleted medium (1× modified M9 medium plus 100 μM 2,2′-dipyridyl and appropriate antibiotics) and/or iron-rich medium (LB broth containing 40 μM FeSO_4_, 0.2 % glucose and appropriate antibiotics), followed by incubation at 30 °C for 16 h with agitation. Cells (100 mg wet weight) from overnight cultures were pelleted by centrifugation at 3000 × g at 4 °C for 30 min and then resuspended in Lysis Buffer (50 mM Tris (pH 8.0), 1 % n-octyl-B-D-thioglucopyranoside, 3 μg/ml DNase I, 3 μg/ml RNase A, 30 μg/ml lysozyme, 1 mM DTT, 1× Protease Inhibitor Cocktail). Whole-cell lysates were incubated on a nutating mixer for 30 min at room temperature and then centrifuged for 5 min at 21,000 × g. Supernatants were recovered for Western blots. Aliquots of cleared cell lysates were separated on 10 % SDS-polyacrylamide gels. Following gel electrophoresis, separated proteins were transferred onto a PVDF membrane using a Mini-Trans Blot Electrophoretic Transfer Cell (Bio-Rad Laboratories). The membrane was blocked for 1 h at room temperature using 5 % skim milk powder in PBST (137 mM NaCl, 2.7 mM KCl, 10 mM Na_2_HPO_4_, 1.8 mM KH_2_PO_4_, 0.2 % Tween 20). Blocked membranes were incubated with one of the following primary antibodies for 1 h at room temperature or at 4 °C overnight: (*i*) mouse monoclonal anti-FLAG antibody (1:1000 dilution; Thermo Fisher Scientific), (*ii*) mouse monoclonal anti-HA antibody (1:1000 dilution; Pierce), (*iii*) mouse monoclonal anti-GAPDH antibody (1:10,000; Thermo Fisher Scientific). Goat anti-mouse conjugated with horseradish peroxidase (HRP) (1: 10,000–1:20,000 dilution; Santa Cruz Biotechnology) was used as a secondary antibody. HRP activity was visualized using a SuperSignal™ West Pico Chemiluminescent Substrate (Thermo Fisher Scientific).

## Results

### Construction of pFCF1, pFCF2 and pFBH1

**Fig. 2:**
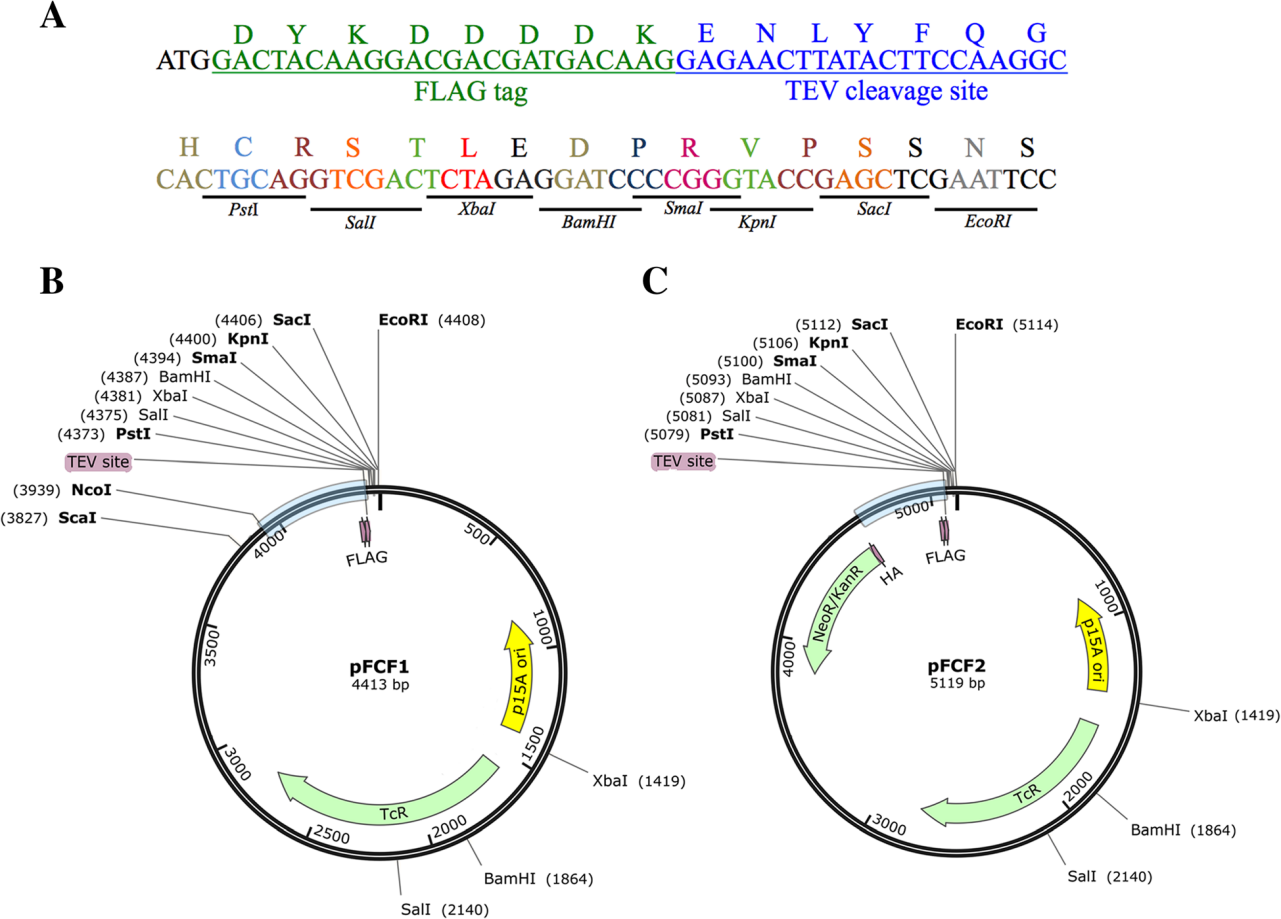
Vector maps of pFCF1 and pFCF2. **a** Polypeptide sequence immediately downstream of the bidirectional promoter region found in gBlock1. Start codon sequence: black, FLAG sequence: green, TEV cleavage sequence: blue. MCS region from pUT18C colored by codons with restriction endonuclease sites shown below sequence. **b** pFCF1 vector map. Light blue bar indicates the promoter region. Unique restriction endonuclease sites shown in bold. FLAG: FLAG tag sequence; TEV: TEV cleavage site; TcR: tetracycline resistance gene; p15A ori: origin of replication. **c** pFCF2 vector map. Light blue bar indicates the promoter region. Unique restriction endonuclease sites shown in bold. HA: HA tag sequence; NeoR/KanR: neomycin/kanamycin resistance gene

**Fig. 3:**
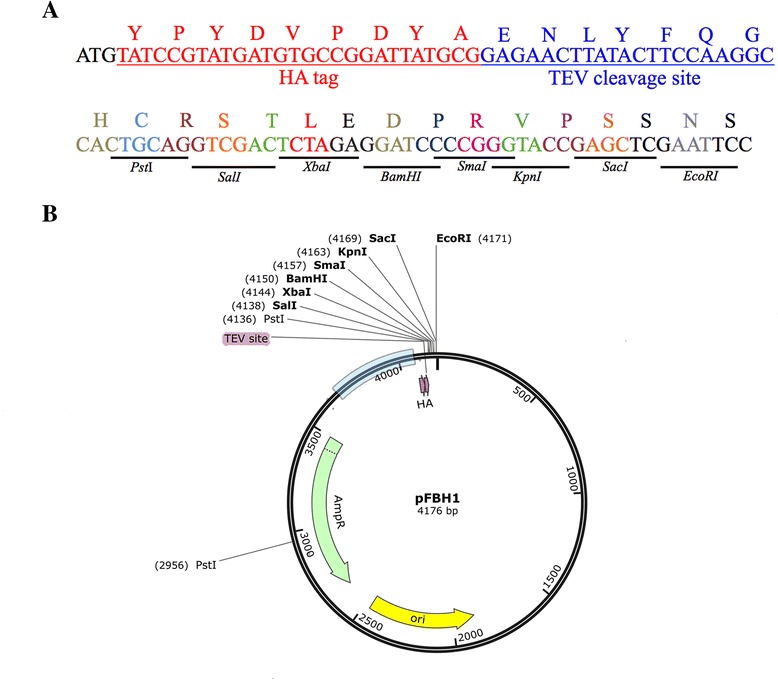
Vector map of pFBH1. **a** Polypeptide sequence immediately downstream of the bidirectional promoter region found in gBlock3. Start codon sequence: black, HA sequence: red, TEV cleavage sequence: blue. MCS region from pUT18C colored by codons with restriction endonuclease sites shown below sequence. **b** pFBH1 vector map. Light blue bar indicates the promoter region. Unique restriction endonuclease sites shown in bold. HA: HA tag sequence; TEV: TEV cleavage site, AmpR: ampicillin resistance gene; ori: pMB1 origin of replication

The vector pFCF1 was constructed by inserting gBlock1 (Additional file 2: Figure S1) into a pACYC184 backbone. gBlock1 encodes the bidirectional Fur promoter region (Fig. 1a) followed by a downstream FLAG epitope tag sequence, TEV protease cleavage site, and a multiple cloning site (MCS) (Fig. 2a). We used the pUT18C MCS sequence (Euromedex) for subcloning of ORFs from BACTH vectors directly into pFCF1. Iron-starved *E. coli* transformants harboring ORFs subcloned into pFCF1 would thus express recombinant proteins with cleavable N-terminal FLAG tags. The map of pFCF1 is shown in Fig. 2b. We also generated pFCF2, a pFCF1-derived vector, to demonstrate bidirectional expression of two proteins from the plasmid-borne *fepB*/*entC* promoter region. To construct pFCF2, we designed gBlock2 (Additional file 3: Figure S2), which contains an in-frame HA tag sequence upstream of the kanamycin resistance gene (*KanR*) that encodes neomycin-kanamycin phosphotransferase II [25]. To generate pFCF2, gBlock2 was synthesized and then inserted between the NcoI and ScaI sites of pFCF1. The map of pFCF2 is shown in Fig. 2c. The vector pFBH1 was constructed by insertion of gBlock3 (Additional file 4: Figure S3) between the EcoRI and SalI sites of linearized pBR322. gBlock3 contained DNA encoding the bidirectional Fur promoter region (Fig. 1a), the HA tag sequence, a TEV protease cleavage site, and the MCS from pUT18C (Fig. 3a). The pFBH1 vector (Fig. 3b) allows for iron-controlled expression of recombinant proteins with cleavable N-terminal HA tags.

### Assessment of iron-responsive promoter regions

**Fig. 4:**
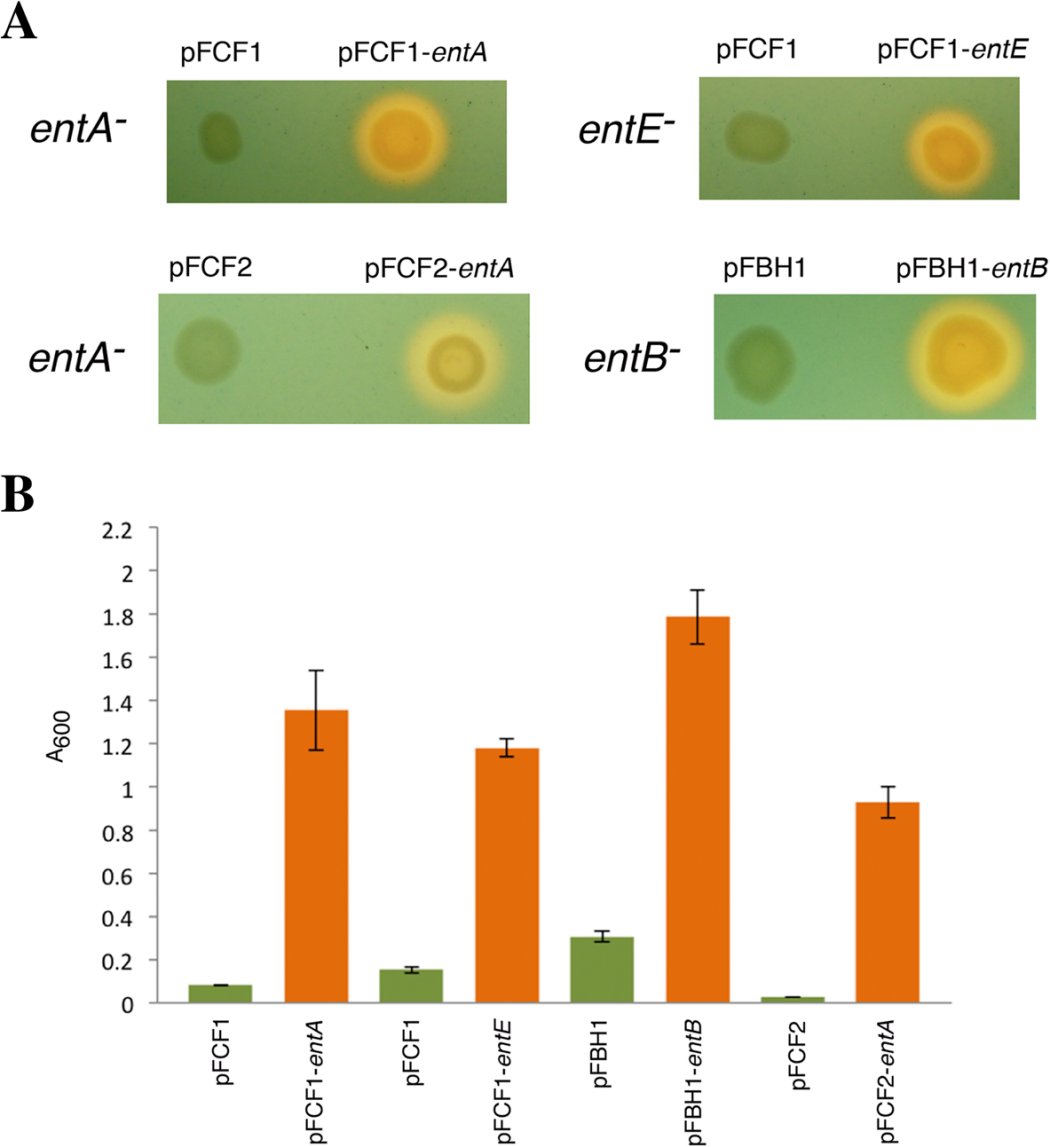
Functional assays for pFCF1, pFCF2 and pFBH1. **a** CAS assays. Images are photographs of CAS agar plates spotted with various *E. coli* transformants. Enterobactin secretion is indicated by orange halos. Upper left panel: *E. coli entA* ^*−*^ strain transformed with pFCF1 (*left spot*) and pFCF1-*entA* (*right spot*). Lower left panel: *E. coli entA* ^−^ strain transformed with pFCF2 (*left spot*) and pFCF2- *entA* (*right spot*). Upper right panel: *E. coli entE* ^*−*^ strain transformed with pFCF1 (*left spot*) and pFCF1-*entE* (*right spot*). Lower right panel: *E. coli entB* ^*−*^ strain transformed with pFBH1 (*left spot*) and pFBH1-*entB* (*right spot*). **b** Growth studies of transformants in iron-depleted medium. Orange bars indicate strains/transformants that exhibited a positive CAS signal; error bars represent standard deviations from mean values (*n* = 3). Left to right: *E. coli entA* ^−^ strain transformed with pFCF1; *E. coli entA* ^*−*^ strain transformed with pFCF1-*entA*; *E. coli entE* ^*−*^ strain transformed with pFCF1; *E. coli entE* ^−^ strain transformed with pFCF1-*entE*; *E. coli entB* ^−^ strain transformed with pFBH1; *E. coli entB* ^*−*^ strain transformed with pFBH1-*entB*; *E. coli entA* ^−^ strain transformed with pFCF2; *E. coli entA* ^*−*^ strain transformed with pFCF2-*entA*

To determine functionality of pFCF1, pFCF2, and pFBH1, we subcloned ORFs encoding *E. coli* enterobactin biosynthetic enzymes into the MCS regions of these vectors and performed complementation experiments using relevant knockout strains. Genes encoding the enterobactin biosynthetic enzymes EntA, EntE, and EntB were prepared by PCR amplification from pCA24N-based constructs as reported previously [21]. Specifically, the *entA* gene was subcloned into pFCF1 and pFCF2 to produce pFCF1-*entA* and pFCF2-*entA*. The *entE* gene was subcloned into pFCF1 to produce pFCF1-*entE*. Finally, the *entB* gene was subcloned into pFBH1 to produce pFBH1-*entB*. The four constructs were transformed into respective *entA*^−^, *entE*^−^ and *entB*^−^*E. coli* knockout strains, and CAS assays [24] were used to assess complementation of the knockout phenotype (i.e., impaired enterobactin biosynthesis). The CAS assay is a classical technique used to detect for the presence of siderophores. Upon iron chelation by siderophores, a color change of a dye complex (from blue/green to orange) is observed. Transformants containing pFCF1-*entA* and pFCF-2 *entA* in an *entA*^−^ background were observed to produce orange halos indicative of iron chelation due to functional enterobactin biosynthesis whereas no halos were observed for empty vector controls (Fig. 4a, upper and lower left panels). Similar results were found for the pFCF1-*entE* transformant in the *entE*^−^ background (Fig. 4a, upper right panel), as well as for the pFBH1-*entB* transformant in the *entB*^−^ background (Fig. 4a, lower right panel). Growth studies (Fig. 4b) were consistent with our CAS assay results. Low growth was observed for *entA*^*−*^ and *entE*^*−*^*E. coli* strains transformed with pFCF1 (Fig. 4b, columns 1 and 3). These knockout strains were rescued by transformation with pFCF1-*entA* and pFCF1-*entE*, respectively (Fig. 4b, columns 2 and 4). An *entB*^−^ strain transformed with pFBH1 also exhibited low growth (Fig. 4b, column 5), while transformation with pFBH1-*entB* complemented the knockout phenotype (Fig. 4b, column 6). Consistent with the above results, the *entA*^−^ strain transformed with pFCF2 did not exhibit significant growth in iron-depleted medium (Fig. 4b, column 7), whereas the pFCF2-*entA* transformant grew well (Fig. 4b, column 8).

### Iron-controlled protein expression

**Fig. 5:**
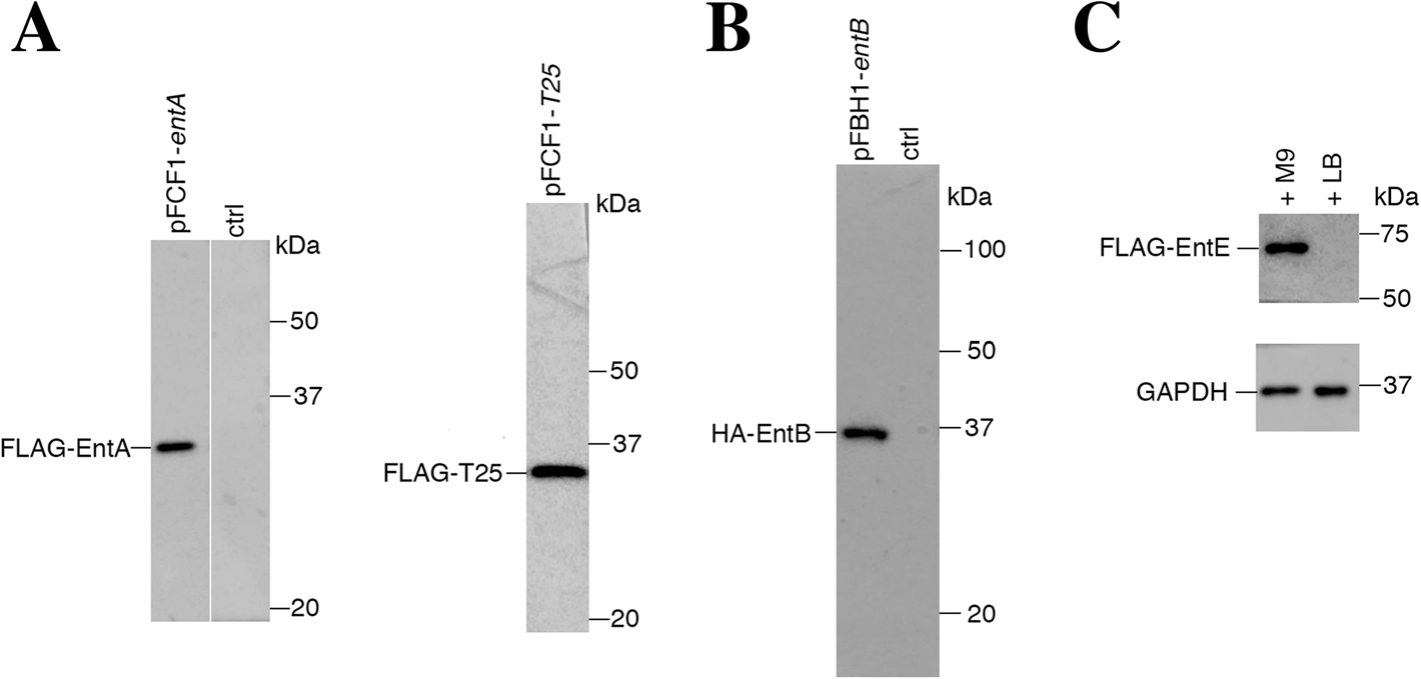
Protein expression from pFCF1 and pFBH1. **a** Whole-cell lysates from iron-starved *E. coli* BW25113 transformants (equivalent wet cell weights) were analyzed by Western blotting. An anti-FLAG antibody was used to detect expression of FLAG-EntA (*left blot, left lane*) and FLAG-T25 (*right blot*). Untransformed cell lysate probed with anti-FLAG antibody (*left blot, right lane (‘ctrl’)*). **b** Western blot of iron-starved *E. coli* BW25113 lysates probed with anti-HA antibody. Left lane: *E. coli* BW25113 transformed with pFBH1-*entB*. Right lane: Untransformed cell lysate (‘ctrl’). **c** Iron responsiveness of the bidirectional promoter region. Upper panel: anti-FLAG antibody was used to detect expression of FLAG-EntE in lysates from cells grown in minimal M9 medium supplemented with 2,2'-dipyridyl (*left*) and from cells grown in LB medium supplemented with FeSO_4_ (*right*). Lower panel: anti-GAPDH antibody was used to detect expression of *E. coli* GAPDH from protein samples identical to those in the upper panel

To investigate iron-controlled protein expression from pFCF1 and pFBH1, we performed Western blotting analysis on isolated soluble proteins from iron-starved *E. coli* cells transformed with pFCF1-*entA* and pFBH1-*entB*. In addition, we used pFCF1 to examine iron-controlled expression of a protein not related to iron metabolism. For this experiment we used the DNA encoding T25, part of the catalytic fragment of adenylate cyclase from *B. pertussis* [26] to produce pFCF1-*T25*. Proteins from whole-cell lysates (equivalent cell wet weights) of transformants were separated by SDS-PAGE and the presence of epitope-tagged recombinant proteins was detected by Western blotting using appropriate antibodies directed against epitope tags. Expression of FLAG-tagged EntA from pFCF1-*entA* was detected using an anti-FLAG antibody (Fig. 5a, left blot, left lane). As a negative control, untransformed lysate was probed with anti-FLAG antibody and no signal was observed (Fig. 5a, left blot, right lane). Epitope signals were also observed for FLAG-tagged T25 expressed from pFCF1-*T25* (Fig. 5a, right blot) and HA-tagged EntB expressed from pFBH1-*entB* (Fig. 5b, left lane). Proteins recovered from untransformed lysate probed with anti-HA antibody resulted in no observable signal (Fig. 5b, right lane).

To determine that the *fepB*/*entC* Fur promoter region was iron-responsive, we grew *E. coli* BW25113 cells transformed with pFCF1-*entE* under iron-rich conditions using LB medium supplemented with FeSO_4_ as well as under iron-restricted conditions using modified M9 medium supplemented with 2,2′-dipyridyl. Cells were recovered from respective overnight cultures and equivalent amounts of proteins from whole-cell lysates were separated by SDS-PAGE. Western blot analysis using an anti-FLAG antibody revealed that FLAG-EntE was only detected in cells grown in iron-depleted medium, whereas no signal was observed from transformants grown in iron-rich medium (Fig. 5c, upper panel). As a control, proteins from identical lysate loadings were probed with an anti-GAPDH antibody. Comparable GAPDH signals were observed in lysates from cells grown in both iron-depleted and iron-rich media (Fig. 5c, lower panel).

### Iron-controlled bidirectional expression from pFCF2

**Fig. 6:**
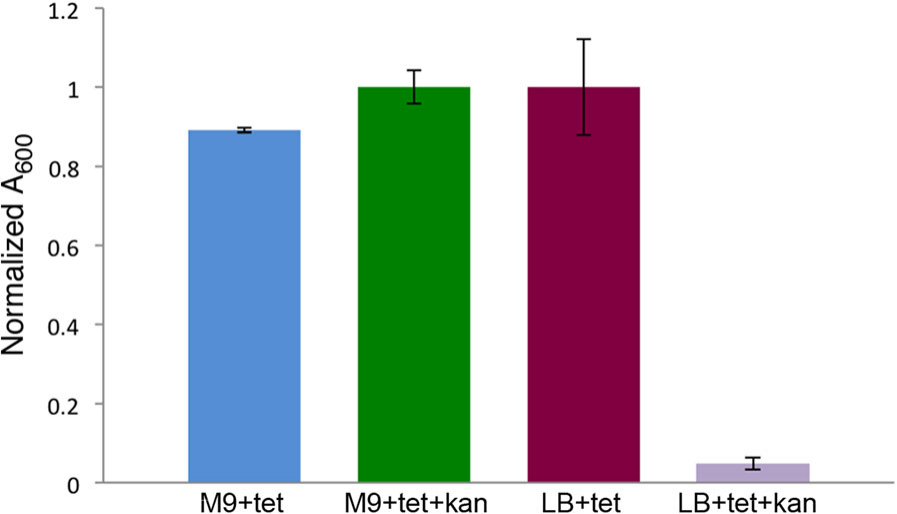
Bidirectional expression from pFCF2. Iron-controlled expression from the anti-clockwise-oriented *KanR* gene in pFCF2 was determined by bacterial growth studies. Shown are normalized cell densities (A_600_) of *E. coli entA* ^−^ strains transformed with pFCF2-*entA* grown in (*left to right*): M9 medium + 2,2'-dipyridyl supplemented with tetracycline; M9 medium + 2,2'-dipyridyl supplemented with tetracyline and kanamycin; LB medium + FeSO_4_ supplemented with tetracycline; LB medium + FeSO_4_ supplemented with tetracycline and kanamycin. Error bars represent standard deviations from mean values (*n*=3)

To test for bidirectional protein expression, we subcloned the *E. coli entA* gene into the MCS of pFCF2, which is under the control of Fur box 2. The pFCF2 vector also contains the *KanR* gene oriented in the opposite direction, under the control of Fur box 1. The resulting pFCF2-*entA* construct was transformed into competent *E. coli* BW25113 *entA*^−^ and transformants were grown in either iron-rich (LB + FeSO_4_) medium or iron-depleted (M9 + 2,2′-dipyridyl) medium in the presence and absence of kanamycin. Growth studies on pFCF2-*entA* transformants revealed that transformants grown in iron-depleted media supplemented with tetracycline grew to similar densities in the presence or absence of kanamycin (Fig. 6, columns 1 and 2). Conversely, transformants grown in iron-rich media supplemented with tetracycline grew poorly in the presence of kanamycin (Fig. 6, columns 3 and 4) due to Fur repression under iron-replete conditions.

## Discussion

We designed and constructed three vectors (pFCF1, pFCF2, and pFBH1) for iron-controlled protein expression from low-copy-number vectors in *E. coli*. For pFCF1 and pFCF2, the plasmid pACYC184 [27] (origin of replication: p15A; copy number ~ 15) was used as a backbone, whereas for pFBH1 the plasmid pBR322 [28] (origin of replication: pMB1; copy number ~ 20) was used. These low-copy-number plasmids were chosen as backbones in order to avoid protein overexpression found in systems employing high-copy-number plasmids, such as the pBAD series of expression vectors that contain the pUC origin of replication (copy number ~ 500–700 [29]) or the pTZ-derived expression vectors [30]. Furthermore, since pFCF1/pFCF2 and pFBH1 have different antibiotic resistance markers along with compatible origins of replication, expression of multiple epitope-tagged proteins from a single co-transformant is possible. For all vectors, gBlock® fragments containing: (*i*) a wild-type *E. coli* Fur-controlled bidirectional promoter region, (*ii*) sequences encoding epitope tags (FLAG or HA), (*iii*) sequence encoding the TEV protease cleavage site and (*iv*) a BACTH-compatible MCS, were designed, synthesized and inserted into respective plasmid backbones (pACYC184 or pBR322).

For iron-controlled expression of epitope-tagged proteins, we used the bidirectional promoter region in the intercistronic space between the *fepB* and *entC* genes in the *E. coli* chromosome (Fig. 1a) [18, 19]. This region, which contains all nucleotides between the *fepB* and *entC* start codons (624511–624884 in *E. coli* K12 MG1655 (NCBI Reference Number: NC_000913.3)), has two Fur box sequences. Fur box 1 controls expression of *fepB* in the anti-clockwise direction whereas Fur box 2 controls expression of the operon containing *entC*, *entE*, *entB*, *entA,* and *entH* genes in the clockwise direction. While Fur box 1 overlaps with its cognate −10/−35 sequences, Fur box 2 occurs downstream of its cognate +1/−10/−35 sequences [18, 19].

In order to test the functionality of pFCF1, pFCF2 and pFBH1, derivative constructs containing ORFs (*entA*, *entE*, and *entB*) under the control of Fur box 2 were prepared. Chromosomal expression of these ORFs in *E. coli* is under Fur control, and they were therefore logical candidates for testing the plasmid-borne bidirectional promoter region in pFCF1/2 and pFBH1. CAS assays and growth studies (Fig. 4) confirmed that the derivative constructs were able to complement respective knockout phenotypes upon iron restriction due to Fur derepression. Western blotting demonstrated that the bidirectional promoter region was controlling expression of epitope-tagged proteins FLAG-EntA and HA-EntB from pFCF1 and pFBH1, respectively. Also, expression of FLAG-T25, a FLAG-tagged *B. pertussis* polypeptide that is typically not under control of *E. coli* Fur, was detected. Taken together, these outcomes demonstrate that the *fepB*/*entC* promoter region (Fig. 1a) is functional in pFCF1 and pFBH1. Experiments using iron-depleted and iron-rich media showed that expression of FLAG-EntE from pFCF1-*entE* occurred in an iron-controlled manner. This is consistent with recent reports demonstrating that elevated expression of genes under Fur control occurred under iron-restricted conditions [10, 31].

Although unidirectional protein expression controlled by Fur box 2 was observed in pFCF1 and pFBH1, the bidirectionality of the *fepB*/*entC* promoter region had to be tested. We designed pFCF2 such that expression of the *KanR* gene would be controlled by Fur box 1 while Fur box 2 would simultaneously control expression of an ORF subcloned into the MCS in the other direction. A pFCF2-*entA* transformant grown under iron-restricted conditions was expected to exhibit complementation of the *entA*^*−*^ phenotype with concomitant kanamycin resistance. Using iron-depleted CAS agar plates supplemented with tetracycline and kanamycin, the pFCF2-*entA* transformant complemented the *entA*^−^ phenotype and produced a CAS halo while being resistant to kanamycin (Fig. 4a, lower left panel). This demonstrated that the *KanR* gene in pFCF2-*entA* was under the control of Fur box 1 resulting in iron-regulated expression. Furthermore, growth studies revealed that the pFCF2-*entA* transformant grew more poorly in iron-rich medium supplemented with kanamycin compared to growth in iron-depleted medium plus kanamycin. This suggests that under iron-replete conditions, Fur-controlled expression of the *KanR* gene is repressed relative to that of the same transformant grown under iron-depleted conditions. Taken together our results demonstrate that bidirectional iron-controlled *in trans* protein expression from pFCF2-*entA* occurs in iron-starved *E. coli* transformants. Protein expression controlled by Fur box 1 in pFCF2 is currently restricted to the *KanR* gene. By replacement of this gene with an additional MCS, bidirectional expression of any two epitope-tagged proteins can be achieved in a single transformant. The compatibility of pFCF- and pFBH-derived constructs would further allow for expression of up to four epitope-tagged proteins in a single co-transformant.

## Conclusions

Constructs containing engineered Fur box sequences for unidirectional expression of toxic genes *in E. coli* have previously been reported [31]. To facilitate bidirectional expression of epitope-tagged proteins under iron control, we have designed and constructed three novel low-copy-number vectors derived from pACYC184 and pBR322. These vectors contain the wild-type intercistronic region found between the *fepB* and *entC* genes in the *E. coli* chromosome that can be used for bidirectional expression. As we have demonstrated, when inserted into low-copy-number plasmid vectors, this region can control simultaneous expression of two proteins in a single transformant. Since the pFCF1/2 and pFBH1 have compatible origins of replication and different antibiotic resistance gene markers, they can also be useful for co-transformation.

Our current understanding of Fur regulation indicates that there are approximately 80 *E. coli* genes under the control of Fur [10]. By transferring ORFs naturally found in Fur regulons, the vectors reported here can be used for a wide variety of experiments such as the study of *in trans* complementation of knockout phenotypes, effects of iron-controlled protein expression on cellular processes (e.g., oxidative stress response, TCA cycle, etc.), as well as studies on proteins that are involved in Fur-controlled virulence mechanisms (e.g., Type 3 secretion system) [5]. Furthermore, genes not typically under Fur control can be expressed in an iron-controlled manner. Since the MCS in pFCF1 and pFBH1 is compatible with the BACTH system, ORFs encoding interacting partners detected by BACTH could be easily subcloned into the vectors reported here. Such constructs could be used for follow-up studies such as co-immunoprecipitation experiments using appropriate antibodies directed against vector-encoded epitope-tagged proteins.

## Additional files

Additional file 1: Table S1. PCR primer sequences used for preparing pFCF1 and pFBH1 constructs. (DOCX 58 kb) Additional file 2: Figure S1. Nucleotide sequence of gBlock1 containing bidirectional Fur promoter region. Black text: Region encoding *E. coli* bidirectional Fur promoter region between *fepB* and *entC*. Red text: NcoI restriction endonuclease site. Green text: EcoRI restriction endonuclease site. Orange text: FLAG-encoding region. Blue text: Region encoding TEV protease cleavage site. Start codon is highlighted in yellow. MCS region from pUT18C is shown in bolded and underlined text. (TIF 192 kb) Additional file 3: Figure S2. Nucleotide sequence of gBlock2 containing kanamycin resistance gene. Black text: Kanamycin resistance gene from pKT25. Red text: Sequence overlapping with pFCF1 region upstream of NcoI restriction endonuclease site. Green text: Sequence overlapping with pFCF1 region downstream of ScaI restriction endonuclease site. Orange text: Region encoding the HA tag. Start codon is highlighted in yellow. (TIF 320 kb) Additional file 4: Figure S3. Nucleotide sequence of gBlock3 containing bidirectional Fur promoter region. Black text: Region encoding *E. coli* bidirectional Fur promoter region between *fepB* and *entC*. Red text: Sequence overlapping with pBR322 upstream of EcoRI restriction endonuclease site. Green text: Sequence overlapping with pBR322 downstream of SalI restriction endonuclease site. Orange text: Region encoding the HA tag. Start codon is highlighted in yellow. Orange text: Region encoding HA tag. Blue text: Region encoding TEV protease cleavage site. Start codon is highlighted in yellow. MCS region from pUT18C is shown in bolded and underlined text. (TIF 202 kb)

## Abbreviations

BACTH: Bacterial adenylate cyclase two-hybrid
CAS: Chrome azurol S
DIP: 2,2'-dipyridyl
DTT: Dithiothreitol
Fur: Ferric Uptake Regulator
GAPDH: Glyceraldehyde 3-phosphate dehydrogenase
HRP: Horseradish peroxidase
IPTG: Isopropyl β-D-1-thiogalactopyranoside
MCS: Multiple cloning site
ORF: Open reading frame
PVDF: Polyvinylidene difluoride
SDS-PAGE: SDS-polyacrylamide gel electrophoresis
