# Supplementary material for: A novel set of vectors for Fur-controlled protein expression under iron deprivation in Escherichia coli
Source: BMC Biotechnol. 2016 Sep 13;16(1):68. doi: 10.1186/s12896-016-0298-1 (PMC5020551; doi:10.1186/s12896-016-0298-1)
Supplement: Additional file 1: Table S1. — PCR primer sequences used for preparing pFCF1 and pFBH1 constructs. (DOCX 58 kb) [file 12896_2016_298_MOESM1_ESM.docx]

**Table S1.** PCR primer sequences used for preparing pFCF1 and pFBH1 constructs.

| Vector | Gene | Primer Sequence (5’-3’) |
| --- | --- | --- |
| pFCF1 | *entA* | F: TAGGGGTACCTATGGATTTCAGCGGTAAAAATGTCTGGG |
|  |  | R: CTACGGAATTCTTATGCCCCCAGCGTTGAGCC |
|  | *entE* | F: TAGGGGTACCTATGAGCATTCCATTCACCCGCTGGC |
|  |  | R: CTACGGAATTCTCAGGCTGATGCGCGTGACG |
|  | *T25* | F:TAGGGGTACCTATGCAGCAATCGCATCAGGCTGGTTACGCAAACG |
|  |  | R: CTACGGAATTCTTAGGCCCGCCGCGTGCGCGCCAGGTAAT |
| pFBH1 | *entB* | F: TAGGGGTACCTATGGCTATTCCAAAATTACAGGCTTACGC |
|  |  | R: CTACGGAATTCTTATTTCACCTCGCGGGAGAGTAGC |
| F = forward, R = reverse. Underlined sequences indicate *Kpn*I (forward) and *Eco*RI (reverse) restriction sites. | | |
