# Supplementary figures and images for: A novel set of vectors for Fur-controlled protein expression under iron deprivation in Escherichia coli
Source: BMC Biotechnol. 2016 Sep 13;16(1):68. doi: 10.1186/s12896-016-0298-1 (PMC5020551; doi:10.1186/s12896-016-0298-1)

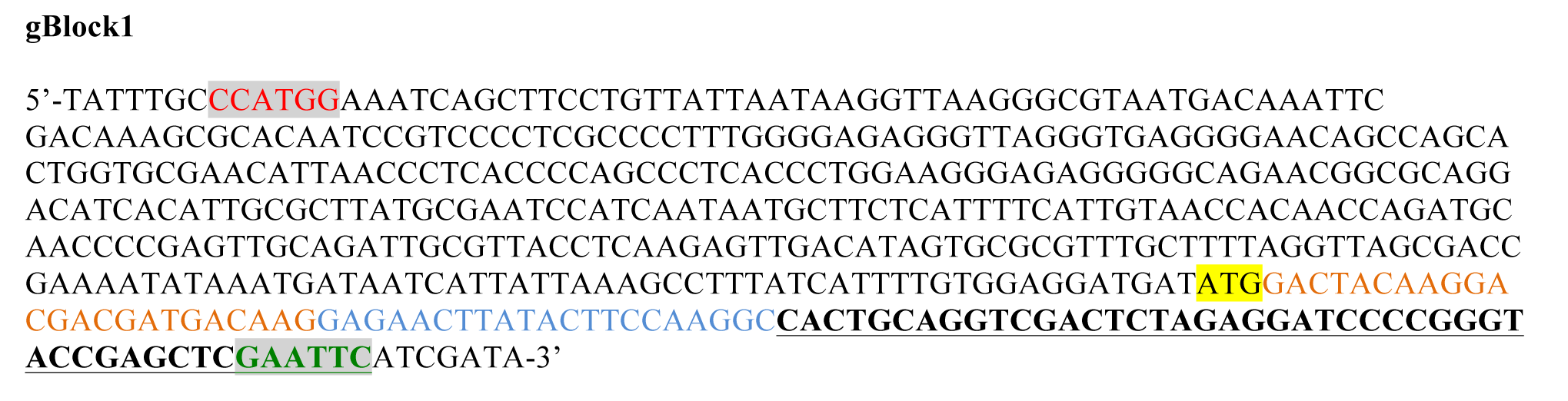

Supplement: Additional file 2: Figure S1. — Nucleotide sequence of gBlock1 containing bidirectional Fur promoter region. Black text: Region encoding E. coli bidirectional Fur promoter region between fepB and entC. Red text: NcoI restriction endonuclease site. Green text: EcoRI restriction endonuclease site. Orange text: FLAG-encoding region. Blue text: Region encoding TEV protease cleavage site. Start codon is highlighted in yellow. MCS region from pUT18C is shown in bolded and underlined text. (TIF 192 kb) [file 12896_2016_298_MOESM2_ESM.tif]

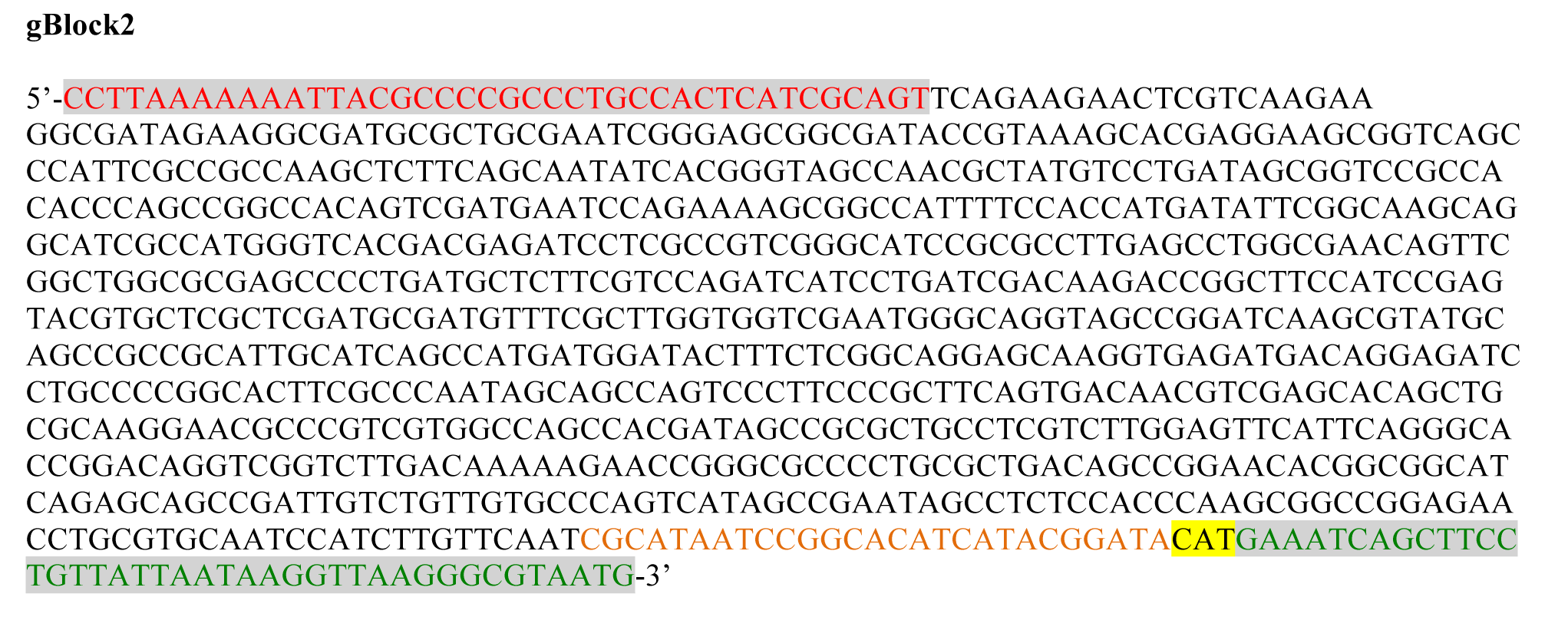

Supplement: Additional file 3: Figure S2. — Nucleotide sequence of gBlock2 containing kanamycin resistance gene. Black text: Kanamycin resistance gene from pKT25. Red text: Sequence overlapping with pFCF1 region upstream of NcoI restriction endonuclease site. Green text: Sequence overlapping with pFCF1 region downstream of ScaI restriction endonuclease site. Orange text: Region encoding the HA tag. Start codon is highlighted in yellow. (TIF 320 kb) [file 12896_2016_298_MOESM3_ESM.tif]

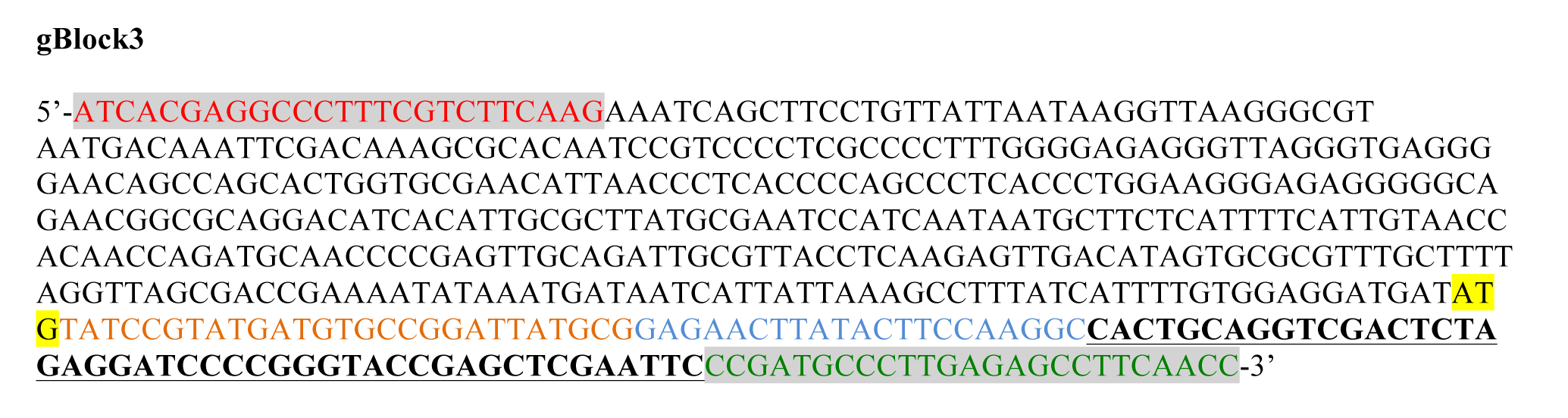

Supplement: Additional file 4: Figure S3. — Nucleotide sequence of gBlock3 containing bidirectional Fur promoter region. Black text: Region encoding E. coli bidirectional Fur promoter region between fepB and entC. Red text: Sequence overlapping with pBR322 upstream of EcoRI restriction endonuclease site. Green text: Sequence overlapping with pBR322 downstream of SalI restriction endonuclease site. Orange text: Region encoding the HA tag. Start codon is highlighted in yellow. Orange text: Region encoding HA tag. Blue text: Region encoding TEV protease cleavage site. Start codon is highlighted in yellow. MCS region from pUT18C is shown in bolded and underlined text. (TIF 202 kb) [file 12896_2016_298_MOESM4_ESM.tif]
